# Ideas evolutivas y raciales en México: el caso de Andrés Molina Enríquez en Los Grandes Problemas Nacionales (1909)

**DOI:** 10.1590/S0104-59702026000100002

**Published:** 2026-03-02

**Authors:** Brian Becerra-Bressant, Ana Barahona

**Affiliations:** i Estudiante de doctorado, Posgrado en Ciencias Biológicas, Facultad de Ciencias/Universidad Nacional Autónoma de México. Coyoacán – Ciudad de México – México. becerra_bressant@ciencias.unam.mx; ii Profesora de tiempo completo, Departamento de Biología Evolutiva/Facultad de Ciencias/Universidad Nacional Autónoma de México. Coyoacán – Ciudad de México – México. ana.barahona@ciencias.unam.mx

**Keywords:** Andrés Molina Enríquez (1868-1940), Charles Darwin (1809-1882), Ernst Haeckel (1834-1919), Razas, Selección y evolución, Andrés Molina Enríquez (1868-1940), Charles Darwin (1809-1882), Ernst Haeckel (1834-1919), Races, Selection and evolution

## Abstract

Este trabajo analiza las ideas evolutivas y raciales de Andrés Molina Enríquez contenidas en su obra *Los grandes problemas nacionales* (1909). En la obra se habla de sociedades superiores e inferiores, razas humanas, atributos indígenas y mestizaje. El escrito propone que dichos temas están atravesados por interpretaciones evolucionistas de Molina Enríquez, basadas en Ernst Haeckel y Charles Darwin. Aquí se elaboran cuatro ideas: fuerzas internas y externas en Haeckel y Darwin; razas, de Darwin a la fisiología; las selecciones individual y colectiva; Molina Enríquez y el evolucionismo mexicano. Con estos apartados, se muestra que el autor estudiado fue un importante evolucionista en el México del Porfiriato.

## Abstract (en)

This paper analyzes the evolutionary and racial ideas of Andrés Molina Enríquez contained in his work Los grandes problemas nacionales (1909). The work explores the concepts of superior and inferior societies, human races, indigenous attributes, and mestizaje. The analysis suggests that these themes are influenced by Molina Enríquez’s evolutionary interpretations, based on Ernst Haeckel and Charles Darwin. Four ideas are elaborated here: internal and external forces in Haeckel and Darwin; races, from Darwin to physiology; individual and collective selection; and Molina Enríquez and Mexican evolutionism. These sections show that the author studied was a significant evolutionist in Mexico during the Porfiriato.

En 1909 Andrés Molina Enríquez (1868-1940) publicó la extensa y compleja obra *Los grandes problemas nacionales* (de aquí en adelante LGPN),^1^ cuya intención fue la resolución de cinco problemas del México de finales del siglo XIX y principios del siglo XX: (1) la propiedad, (2) el crédito territorial, (3) la irrigación de aguas, (4) la población, y (5) la política (Molina Enríquez, 2016, p.131, 199, 255, 307, 417). Sobre ese eje, se insertan planteamientos particulares: el papel de las razas humanas, las sociedades superiores e inferiores, el mestizo mexicano, la condición de los indígenas o la formación de una patria verdadera, que en este escrito se propone están atravesados por diversas interpretaciones y adaptaciones evolucionistas de Molina Enríquez. El objetivo principal de este texto es explicar esos planteamientos y evidenciar que el autor se vio influenciado por naturalistas como Charles Darwin (1809-1882) y Ernst Haeckel (1834-1919). Asimismo, se busca mostrar que Molina poseía conocimientos, lecturas e interpretaciones evolucionistas, y que contaba, además, con una propuesta social darwinista que no encajaba con la praxis tradicional de la época. Diferente a la opinión de otros trabajos, el nuestro sostiene que el autor estudiado podría haber tomado nociones de Darwin para respaldar en LGPN parte de su pensamiento.^2^ También se evidencia que el evolucionismo tuvo un fin político en Molina, ya sea para justificar el mestizaje ideal o para sostener que los indígenas eran los individuos físicamente superiores del territorio nacional. De manera que este texto, por un lado, profundiza en ideas poco examinadas de Molina y, por el otro, vincula posturas historiográficas recientes para realizar futuros estudios sobre cómo se empleó y circuló el evolucionismo durante el Porfiriato (1876-1911).

Se plantean cuatro apartados para desarrollar nuestra propuesta: en el primero, “Fuerzas internas y externas: Haeckel y Darwin”, analizamos la impronta que dejaron ambos autores en los tipos de fuerzas que fundamenta don Andrés en LGPN, una que ocurre dentro del organismo y otra que yace en el ambiente; en el segundo, “Razas: de Darwin a la fisiología”,^3^ estudiamos con detalle las distinciones y agrupaciones raciales articuladas por Molina, las cuales, a nuestro juicio, cuentan con una base de ideas del célebre naturalista inglés; en el tercero, titulado “Las selecciones individual y colectiva”, conjuntamos los fundamentos evolutivos y raciales para ilustrar las rutas de selección molinianas y el advenimiento del mestizo mexicano; y en el cuarto, denominado “Molina Enríquez y el evolucionismo mexicano”, resaltamos, por una parte, los corolarios evolucionistas desarrollados por los coetáneos de Molina y, por la otra, la circulación de este tipo de ideas durante la época. Para finalizar, agregamos un apartado de consideraciones finales.

Hemos encontrado que las ideas de Molina no son una calca de las tesis de Darwin o de Haeckel. A finales del siglo XIX y principios del siglo XX era complejo hacer una interpretación exacta de las teorías evolucionistas. De hecho, los naturalistas se leían entre ellos y readaptaban sus teorías, como el caso del propio Haeckel interpretando a Darwin.^4^ En ese sentido, Molina también reconceptualiza nociones y términos provenientes de *El origen de las especies* o *La historia de la creación*. No obstante, el conocimiento y la influencia de autores naturalistas en Molina también son evidentes, marcados y amplios.

Antes de pasar al análisis de estos apartados, es importante mencionar que Molina Enríquez nació en Jilotepec, México, en una villa habitada por criollos y mestizos, pero rodeada de comunidades indígenas. A temprana edad ingresó al Instituto Literario del Estado de México, que contaba con un plan de estudios basado en el positivismo (Shadle, 1994, p.14; Kourí, 2009, p.286) y con un modelo de educación científica similar al plasmado por Gabino Barreda en la Escuela Nacional Preparatoria (Guevara, 2006, p.228).^5^ Tras educarse en el Instituto, el adolescente Molina tenía una sólida formación positivista, que respondía al apogeo científico del Porfiriato (Azuela, 1996, 2018). De 1885 a 1891 estudió en la Escuela Nacional de Jurisprudencia y posteriormente fue notario en Jilotepec, Toluca y Sultepec, lugares donde corroboró la privatización de tierras indígenas.^6^ Los orígenes positivistas y la transacción de tierras comunales a finales del siglo XIX marcarían el pensamiento de nuestro autor, pues, tanto su educación científica, como las experiencias en torno a las tierras de los “indios”^7^ serían la base de la obra que nos atañe (Kouri, 2009, p.289; Castañeda, 2001, p.194-195). A inicios del siglo XX, Molina ejerció la abogacía en la Ciudad de México y escribió textos en su periódico *La Hormiga* (1898), publicó *La reforma y Juárez* (Molina Enríquez, 1906) y redactó artículos en el diario *El Tiempo* (1906), que en su conjunto vislumbraban el contenido de LGPN (1909).

La figura y la obra de Molina han sido estudiadas desde diversos enfoques, por ejemplo: Bonfil Batalla (1967) y Comas (1966) resaltan las reflexiones del autor a favor de los indígenas; Brading (1988) recalca el rol que desempeñaron LGPN en la construcción del Estado mexicano; Shadle (1994) analiza la posición de don Andrés con respecto a las tierras mexicanas; Basave (1992, 2001) y Krauze (8 mar. 1998) revisan la “mestizofilia” del abogado; y Kourí (2009) presenta una nutrida compilación con motivo del centenario de LGPN. De nuestro mayor interés, por discutir la visión de Molina sobre las razas, son los estudios de Moya (2015) y Magallón (2003). Asimismo, porque tratan el evolucionismo de Molina, resaltan Juárez-Barrera y Bueno-Hernández (2017), quienes sugieren concepciones lineales y laxas de evolución, hechas por intelectuales mexicanos, para apoyar el mestizaje; y Corona y Argueta (2017), que sucintamente describen fenómenos evolutivos elaborados por Molina, considerado ecléctico en el debate racista del México decimonónico.

## Fuerzas internas y externas: Haeckel y Darwin

Desde que comienza la defensa de LGPN, Molina Enríquez recubre sus ideas políticas y mestizantes con un velo de conocimientos evolutivos y raciales. Los estudios sobre la naturaleza y afines no le son extraños; tampoco le son ajenos autores como Darwin o Haeckel. La incidencia del segundo en Molina es notoria e interesante.^8^

Con el propósito de analizar los efectos de esta influencia, revisamos los dos tipos de fuerzas que para Molina rigen el curso de la vida: las fuerzas internas, de acción, y las fuerzas externas, de resistencia. La fuerza de acción es también llamada “fuerza formatriz interna”, concepto empleado originalmente por Ernst Haeckel (1876). Cuando Molina abunda en que existen, “en el proceso físico-químico de la vida, las fuerzas interiores que por efecto de la combustión vital se desarrollan en cada uno de los organismos, fuerzas que en conjunto llamó Haeckel fuerza formatriz interna (*Historia de la Creación Natural*)” (Molina Enríquez, 2016, p.63), se establece una correspondencia directa con el naturalista y su “fuerza vital misteriosa, de un poder activo y propósito definido, que está fuera de la materia y que toma las fuerzas físico-químicas a su servicio” (Haeckel, 1876, p.334).^9^ La lectura de la fuente primaria permite a Molina ejecutar una interpretación de las fuerzas internas que, en términos menos abstractos, para nosotros alude a los fenómenos que ocurren dentro del cuerpo humano; a todo lo que sucede antes de nuestra barrera física, la piel.

En cambio, los fenómenos que ocurren más allá de nuestra limitación corpórea corresponden a las fuerzas de resistencia. Estas fuerzas pueden denominarse, como dice Molina Enríquez (2016, p.63), “exteriores o ambientes”, y son “la gravedad, la presión atmosférica, el clima, la humedad etcétera”. En ese sentido, si el ambiente participa, las nociones de Darwin podrían estar implicadas en la configuración de estas fuerzas. El modelo expuesto en *El origen de las especies* (Darwin, 1859) integra el papel del ambiente como una causa generadora de variación, relacionado a las ideas de uso y desuso y herencia de caracteres adquiridos. El dato se puede corroborar en los capítulos I, “Causas de la variabilidad”, y V, “Efectos del cambio de condiciones”. Darwin (2019, p.7-8) apunta en estas secciones, similar a como lo haría posteriormente Molina, que “los seres orgánicos deben estar expuestos durante varias generaciones a condiciones nuevas para que se produzca un grado considerable de variación; y una vez que el organismo ha empezado a variar, generalmente sigue variando por muchas generaciones”. De manera que “todos estos cambios de estructura”, sigue Darwin, “que aparecen entre muchos individuos que viven juntos”, son “efectos indefinidos de las condiciones de vida sobre cada organismo” (p.109). Si bien es cierto que Molina no recupera exactamente el papel del ambiente descrito por Darwin, podría haberlo tomado como referencia para elaborar sus tesis. En LGPN, el autor menciona un par de veces *El origen de las especies* y algunas otras ocasiones cita *El origen del hombre* (de 1871) y *La variación de los animales y las plantas bajo domesticación* (de 1868), lo cual sugiere una lectura del positivista a distintos escritos que componen la obra del naturalista.^10^ Estos acercamientos, que no parecen profundos, pero tampoco superficiales, muestran la apuesta de Molina por un pensamiento de tipo evolucionista.

Conviene resaltar, de las fuerzas molinianas, que “la acción de las primeras y la resistencia de las segundas determinan en su equilibrio lo que pudiéramos llamar la arquitectura de los organismos” (Molina Enríquez, 2016, p.63). ¿A qué se refiere don Andrés con equilibrio y arquitectura? A choques constantes, en distintas intensidades, entre la acción y la resistencia, que dan como resultado la “forma” de los organismos, cuyo desarrollo es similar ante iguales condiciones externas. En otras palabras, si hay fuerzas externas permanentes (clima y humedad invariables, idéntica alimentación, mismas condiciones ambientales), que generen nula o poca alteración en la resistencia, entonces las fuerzas de acción, la “fuerza formatriz interna”, se estabiliza y produce formas y organismos similares. Sin embargo, cuando fuerzas ambientales son distintas (modificación del clima, de la presión o la alimentación), la resistencia varía. Entonces las fuerzas internas sufren estímulos de adaptación, por ende, se genera reacomodo de la fuerza formatriz y se sufren cambios de dirección y nuevos impulsos, provocando formas variadas y la constitución de individuos diferentes:

Como la fuerza formatriz interna es de acción, es en su esencia susceptible de variar según las resistencias, y es claro que, si las resistencias opuestas por las fuerzas ambientes exteriores son continuas y permanentes, dicha fuerza formatriz acabará por producir en todos los casos, formas relativamente iguales. Por el contrario, si las resistencias continuamente varían, la fuerza formatriz, en su trabajo de acomodarse a ellas, se verá obligada a cambiar frecuentemente de dirección, y las formas resultantes tendrán que ser muy variadas (Molina Enríquez, 2016, p.64).

**Figura 1:**
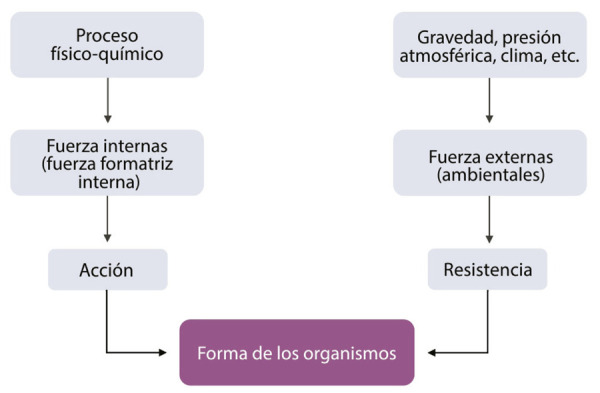
: Esquema que explica las rutas que siguen las fuerzas internas y externas (Fuente: elaboración propia)

**Figura 2:**
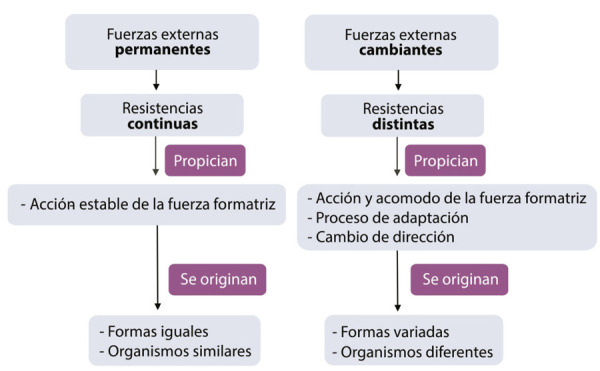
: Esquema que explica las formas resultantes de los organismos de acuerdo con las fuerzas exteriores (Fuente: elaboración propia)

En función de lo anterior, proponemos y trazamos las rutas que siguen ambas fuerzas molinianas: por un lado, los fenómenos físico-químicos se procesan por acción de la fuerza formatriz; por el otro, las fuerzas ambientales provocan resistencia en los individuos. Ambas fuerzas determinan la forma de los organismos (Figura 1), cuya modificación dependerá de la permanencia o la variación de las fuerzas ambientales o exteriores (Figura 2).

Examinamos cómo se desglosa una interesante explicación sobre la evolución orgánica y la variación de formas cuando el ambiente cambia. Impera un sistema cuyas fuerzas internas producen acción, y fuerzas externas producen resistencia. La interacción de ambas fuerzas otorga forma a los organismos. Por forma, parece que Molina se refiere a la constitución anatomofisiológica de los organismos, el cómo se ven y el cómo se estructuran anatómicamente.

Los fenómenos internos se desencadenan por efectos externos y ambientales. Si no hubiese presiones externas, las formas están condicionadas a permanecer estables. Si las causas externas son las que hacen variar a los organismos, algunas tesis de Darwin resultan *ad hoc* en el modelo evolutivo que propone Molina. Así, es factible que el jilotepense leyera los primeros capítulos de *El origen de las especies*, donde precisamente se abunda sobre la influencia que las condiciones de vida ejercen en la variación de los organismos.^11^A la luz de esto, creemos que la preponderancia del ambiente en Molina es patente, ya que justifica el peso de las condiciones externas para dar forma a los organismos.

La idea de Molina sobre el medio como agente de cambio se puede reforzar desde más puntos de vista. Las elaboraciones previas son similares a las que se localizan en la obra de Haeckel, pero pertenecen al “distinguido” y “gran” Jean-Baptiste Lamarck (1744-1829) (Haeckel, 1876, p.28, 150). El naturalista alemán comenta que *Filosofía zoológica* (Lamarck, 2017 [publicado originalmente en 1809]) es el primer trabajo que expone la teoría de descendencia y adaptación, y para evidenciarlo dedica sendos pasajes en *The history of creation* (p.100-125), de los que Molina pudo haber reflexionado. En el escrito del francés se expone que las diferentes condiciones de vida influyen en la organización y la forma de los organismos, así como en el uso o desuso de sus estructuras; y que la adaptación y transformación se explican por la herencia de caracteres y por el empleo de órganos (p.112-115). De acuerdo con Haeckel y su veta lamarckiana, existen tanto la influencia externa de las condiciones adaptativas de vida, como la reacción interna (hábitos y uso de órganos) del organismo que se adapta a esa condición de vida. Sin embargo, el hecho esencial es que la influencia externa siempre será predominante para que ocurra el cambio en los organismos (p.234, 235). Las ideas de Lamarck que Haeckel recupera en sus textos, aunque no explícitas en Molina, vinculan a LGPN con el conocimiento evolucionista de origen francés. La aparición de la vena lamarckiana, a través de la figura preponderante de Haeckel, revela que las fuerzas molinianas guardan ciertos paralelismos con la literatura científica inglesa, alemana y francesa de la época.

## Razas: de Darwin a la fisiología

Para Molina, los caracteres raciales son una “consecuencia de las circunstancias de la adaptación de los grupos humanos a la zona territorial en que viven” (Molina Enríquez, 2016, p.64-65), donde no hay una fijeza absoluta, pero sí una adaptación a esas condiciones. El autor razona que las razas son entes físicos, estables, pero asimismo variables, cuyo desarrollo y tipificación dependen del ambiente que habitan. Las razas, en sus palabras, “son un conjunto de hombres que por haber vivido largo tiempo en condiciones iguales de medio, han llegado a adquirir cierta uniformidad de organización, señalada por cierta uniformidad de tipo” (p.65). Las conjeturas del autor nos indican que el medio crea a las razas, y éstas, en nuestra interpretación, participan sucesivamente de mejores adaptaciones, sujetas irremediablemente a las volátiles condiciones de existencia y a la variación que sufren los individuos.

Si existen las razas, ¿cuáles son las competencias entre ellas? De acuerdo con Molina, la selección de los grupos humanos actúa según los límites geográficos, las relaciones familiares y la búsqueda de alimento. A propósito de la alimentación, el autor toma parcialmente la teoría del carbón y la fuerza vital propuestas por Haeckel (1876, p.329-330), que explican el fenómeno de la vida como producto de las propiedades físicas y químicas del carbono; a partir de él, se combinan otros elementos para sostener los fenómenos de orden vital.^12^ Con base en Haeckel, Molina creía que la absorción del carbono explicaba parte de la evolución orgánica, es decir, la nutrición jugaba un papel clave para desarrollar la fisiología individual y una organización social.^13^ A propósito de los límites geográficos, el abogado emplea los conceptos de adaptación y supervivencia del más apto, provenientes de Darwin (Molina Enríquez, 2016, p.65-66), para sustentar, desde nuestro punto de vista, la selección que atraviesan los grupos humanos cuando rebasan sus confines territoriales.

La idea anterior indica que, si los grupos humanos no llegan a sus límites fronterizos, no hay una competencia; pero si alcanzan los límites, se desprende una activa selección en busca de los más aptos y el mejoramiento del grupo, como lo expresa claramente Molina:

Pero la selección de tal modo perfecciona a todos los organismos, como lo demostró Darwin (*Origen de las especies*), que las unidades de un grupo van saliendo de su zona propia, y en luchas porfiadas con sus vecinas las ocupantes de otras zonas acaban muchas veces por vencerlas y por dilatar su dominio en el territorio de las últimas, no sin sufrir en sí mismas profundas modificaciones (Molina Enríquez, 2016, p.66).

A juicio nuestro, se trata de una adaptación de la selección natural de Darwin, combinada con los conceptos de acción y selección molinianos, y con nociones de autores como Haeckel y Spencer. La influencia de Darwin, en Molina, cobra sentido cuando los organismos salen de su zona y chocan con otras unidades para dominar territorios. En esa pugna, alguna agrupación cae vencida, pero sobrevive, obteniendo modificaciones adaptativas para la siguiente lucha.

Ahora bien, Molina describe tres tipos de raza en México: criollos, mestizos e indígenas. Estos últimos, enuncia el positivista, “son de una antigüedad remotísima y están compuestos de unidades de una poderosísima fuerza racial”, misma que proviene de un largo proceso selectivo “que llegó a producir en el organismo humano de las unidades de esos mismos pueblos, diferencias tan notables con respecto a las demás unidades de la especie” (Molina Enríquez, 2016, p.399). Por lo tanto, “si el objeto y fin de toda selección orgánica es lograr hasta donde sea posible la adaptación al medio”, para Molina “no cabe duda en que el organismo del indio es un organismo superior, como verdaderamente lo es” (p.399).

A partir de lo expuesto, Molina simpatizaba claramente con los indígenas, y nos consta que muchas de esas declaraciones son producto de la influencia que ejerció en él Vicente Riva Palacio (1832-1896). Pasajes enteros de la emblemática obra *México a través de los siglos*, de 1884, del general mexicano se encuentran plasmados en LGPN (veinte páginas: 389-409) para explicar lo que Molina llama fuerza étnica de los elementos indígena y mestizo de nuestra población. Aunque atinadamente se ha dicho que el esquema racial de Molina, “basado en *México a través de los siglos*”, responde a la mezcla de europeos, asiáticos e indígenas para dar lugar a una raza distintiva por su incomparable adaptación al medio (Tenorio, 2009, p.43; Moya, 2015), nosotros concordamos más con los estudios que señalan el influjo de Riva Palacio en Molina para concluir que los indígenas tenían una mayor evolución individual, respecto de los europeos (Sánchez-Rivera, 2019, p.27).

Pero Molina no sólo se vale de Riva Palacio para justificar el cuerpo de los mexicanos, también recurre al doctor Daniel Vergara-Lope (1865-1938). En 1890, trabajando en el Instituto Médico Nacional, Vergara-Lope refutó al médico francés Denis Jourdanet y a su “fisiología de las alturas”, la cual argumentaba que, en México, como resultado de la baja presión atmosférica y la poca saturación de oxígeno en grandes altitudes, los habitantes de esas zonas respiraban un “aire enrarecido” que los hacía susceptibles a enfermedades y limitaba sus capacidades intelectuales (Cházaro, Rodríguez de Romo, 2006). Vergara-Lope no sólo desarrolló una fisiología experimental que invalidaba las tesis de Jourdanet, también sostuvo que el “aire enrarecido” era curativo, y por eso intentó institucionalizar la aeroterapia, eliminando los efectos nocivos del aire y devolviendo la normalidad orgánica a los habitantes (Serrano, 2023, p.77). Así, el doctor reconoció la variación biológica humana como resultado de la aclimatación al medio y contribuyó a desacreditar la inferioridad de las razas en México. Aclimatarse a las alturas, aumentar las respiraciones o ampliar el pecho, no eran modificaciones raciales, sino adaptativas (Cházaro, Rodríguez de Romo, 2006). En ese sentido, Vergara-Lope demostró que el cuerpo de los mexicanos no era inferior por vivir en grandes alturas y defendió las condiciones normales de la raza mexicana.

Molina se apoya tanto en Riva Palacio para sostener “características evolucionistas”, como escasez de barba y vello corporal o ausencia de caninos y muelas del juicio, que otorgan perfección y progreso corporal superior a indígenas y mestizos, como en Vergara-Lope, quien no concuerda con la falta de dientes en los indios, pero sí en que los pobladores de América son lampiños (Molina Enríquez, 2016, p.399). Estos autores respaldan la visión de Molina sobre las razas, pero con matices complementarios: con Riva Palacio, sustenta las ventajas del cuerpo indígena y con Vergara-Lope, la normalidad fisiológica de los mexicanos. Cuando Molina enuncia las cualidades indígenas, no lo hace desinformado, pues argumenta a partir de lo que publican sus connacionales, de quienes analiza (y critica) sus estudios en francés. Además, estos autores le permiten desacreditar la inferioridad indígena e infundir la idea de que las razas serán parte de un mestizaje. Lo anterior quiere decir que, si bien Molina recibió una influencia directa de Riva Palacio en apoyo a los indígenas, también leyó a distintos autores mexicanos para corroborar y darle sustento científico a sus ideas, aunque, al final, prevaleció la superioridad indígena propuesta por el militar mexicano.

## Las selecciones individual y colectiva

En consonancia con el análisis de superioridad expuesto previamente, Molina resalta que sólo el indígena se transporta y se reproduce en territorios hostiles, ya que se adapta a las diferencias de altitud y clima, así como puede vivir de maíz, frijol y chile y vence a las enfermedades y a la muerte. Por esto, declara que “si las razas blancas podían considerarse superiores a las indígenas por la mayor eficacia de su acción, consecuencia lógica de su más adelantada evolución, las razas indígenas podían considerarse como superiores a las razas blancas por la mayor eficacia de su resistencia, consecuencia lógica de su más adelantada selección” (Molina Enríquez, 2016, p.400). Pero entre razas de acción y resistencia, ¿cuáles son realmente superiores? La respuesta es demoledora: “indudablemente las de resistencia”, porque “la raza española” sucumbió frente al desarrollo de los mestizos y sus energías indígenas (p.400-401).

Si bien Molina se inclina por las razas de resistencia, para esclarecerlo argumenta una última idea evolutiva: la selección, que puede ser individual o colectiva. La primera es resultado de la lucha de los individuos dentro de un grupo y “por fuerza produce la progresiva supervivencia de los más aptos” para el acomodo a las condiciones naturales del medio (Molina Enríquez, 2016, p.404-405). El argumento es similar al que se detalla en el capítulo IV de *El origen de las especies*: previa variación, los organismos más aptos son aquellos que se han podido adaptar a las condiciones del medio. Darwin (2019, p.105) reflexiona que cuando surgen variaciones útiles, los ejemplares que las poseen tendrán más posibilidades de vencer en la lucha por la vida, y debido al principio de la herencia, producirán descendientes caracterizados de la misma manera. A este proceso de preservación, donde sobreviven los más aptos, lo llama selección natural. Evidentemente la idea no es calcada tal cual, pues Molina no habla de una variación útil, pero se respeta la esencia principal: tras la adaptación al medio, los más aptos son seleccionados. A nuestro modo de ver, no sería tan cuestionable que esas ideas de Darwin influyeran en el jurista para desarrollar sus fenómenos de selección. Como lo menciona Laura Moya (2015, p.8), el de Jilotepec “retoma la teoría darwinista sobre la evolución advirtiendo la existencia de dos tipos de procesos conjuntos en el desarrollo de todo ser vivo”. Estos procesos justifican, individualmente, “la sobrevivencia de los más aptos” y, colectivamente, “la subsistencia de los grupos sociales más aptos” (p.8).

En cuanto a la selección colectiva, consiste en la lucha de un grupo con otro para determinar los colectivos más aptos y superiores, favoreciendo la vida social y restringiendo la libertad personal. En consecuencia, la selección individual “degenera” cuando opera la selección colectiva, pues ésta, si bien avanza rápidamente “produciendo tipos de raza de muy altas condiciones de evolución superorgánica”, genera “tipos de raza de muy débiles condiciones de adaptación al medio” (Molina Enríquez, 2016, p.406).

Ejemplos de cada selección son China y Francia. En los asiáticos, por sus siglos de aislamiento, predomina la selección individual, con razas fuertes, numerosas, adaptadas al ambiente, pero débiles en evolución colectiva. En los europeos, debido a las incesantes luchas de selección colectiva, abundan los tipos de “perfección” humana, carentes de selección individual y proclives a disminuir su población.

En América, por el aislamiento de un grupo con respecto a otro en el continente, los indígenas difícilmente generaron una selección colectiva. El fenómeno ocurriría tardíamente, en la época moderna, cuando los grupos se aglomeraron y chocaron en la región ístmica; pero mientras esto no pasó, la selección individual “duró larguísimo periodo de tiempo y formó, como era natural, unidades de raza de una fuerza colosal de adaptación” (Molina Enríquez, 2016, p.407-408). En Europa el proceso fue opuesto: los primeros pueblos se aglomeraron alrededor del Mediterráneo, en regiones estrechas, y los choques entre grupos limitaron la selección individual para incrementar la selección colectiva, por lo que el autor de LGPN asevera: “Las razas de resistencia son más fuertes que las razas de acción. En el choque de dos razas rara vez deja de producirse la mezcla de ellas, y en el producto intermedio, a nuestro juicio, domina, como lo indica el señor Riva Palacio, la sangre de la raza más resistente” (Molina Enríquez, 2016, p.409).

Molina concluye que la sangre india predominará en el proceso de mestizaje. En este nuevo mestizaje se imponen los caracteres de los indígenas, que han sido seleccionados a través de los siglos gracias a su resistencia, a su biología y a su sangre combativa. Los indígenas, de selección física y adaptación ambiental, detuvieron su evolución social. En la polaridad de resistencia física biológica y máximo desarrollo social, para Molina los indígenas eran superiores.

**Figura 3:**
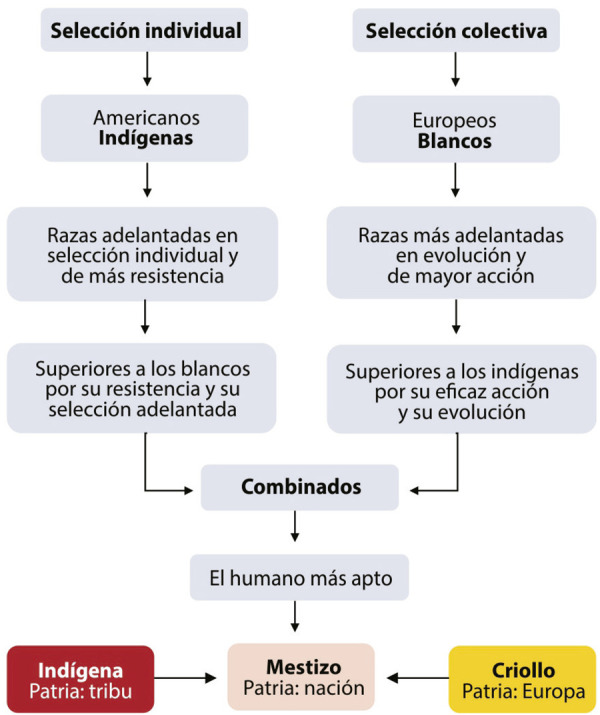
: Esquema con los pasos de las selecciones para llegar al mestizo y al ideal patriótico (Fuente: elaboración propia)

México no es ninguna excepción, y su “raza mestiza no es, en suma, más que la raza indígena modificada ventajosamente por la sangre española”, donde dominan “las características de la muy avanzada selección”. Indígenas y mestizos son superiores a cualquier otra raza “por las condiciones de su incomparable adaptación al medio, por las cualidades de su portentosa fuerza animal”, por “la sujeción absoluta a la alimentación de maíz” y por “la costumbre de vivir casi a la intemperie” (Molina Enríquez, 2016, p.409). Molina encuentra en el mestizo el grupo más apto, por su deseo apoteósico de patria verdadera: la auténtica nación mexicana, aquella cuyos ideales no son ni los de las sociedades occidentales ni los de las tribus indígenas americanas (Figura 3).

Finalmente, vemos cómo el ideal patriótico, basado en el mestizo, es el broche de oro para nuestro autor. La consagración del mestizaje propicia la solución de los grandes problemas nacionales y la consolidación de la patria. En esa tesitura, Molina juzga que los indígenas tenían como patria a sus tribus; que los criollos anhelaban una patria europea; y que los mestizos podían garantizar el futuro del país, por sus dotes para integrar al nativo y al extranjero. De hecho, pareciera que el abogado propone una suerte de fases para alcanzar la máxima etapa: la patria verdadera, cuya realización, indefectiblemente, será a través del mestizo. No es objeto de este estudio profundizar en una “mestizofilia” o en el ideal patriótico de Molina, son temas que han sido abordados por otros autores (Comas, 1966; Basave, 1992; Lomnitz, 2009), en cuyos trabajos es necesario ahondar para entender la posición de don Andrés y para proponer nuevas posturas historiográficas.

## Molina Enríquez y el evolucionismo mexicano

Para hablar de las primeras referencias a Darwin en México, que provinieron de fuentes francesas, nos podemos remontar a los años 1870, 1871 y 1872, cuando, en distintas editoriales y periódicos (*El Libre Pensador*, *El Monitor Republicano*, *El Siglo Diez y Nueve*), diversos autores se refirieron al origen natural del humano y a la lucha por la supervivencia, así como a las críticas de *El origen del hombre* (Ruíz, Noguera, Rodríguez, 2014, p.106-107). Sin embargo, las polémicas iniciales sobre el pensamiento evolucionista ocurrieron en 1877, cuando en la Sociedad Metodófila Gabino Barreda se discutieron los principios de herencia, adaptación y lucha por la existencia (Moreno, 1984, p.45-55; Ruíz, 1987, p.47-75), o en 1878, cuando los hermanos Santiago (1850-1880) y Justo Sierra (1848-1912) debatieron contra periódicos católicos el origen animal o natural del ser humano (Ruíz, Noguera, Rodríguez, 2014). También naturalistas mexicanos fueron parte de discusiones importantes, como José Ramírez (1852-1904) o Alfredo Dugès (1826-1910), quienes mostraron su conocimiento sobre las ideas de Haeckel y Lamarck, no sólo las de Darwin (Ruíz, Noguera, Rodríguez, 2014; Barahona, 2009). Estos sucesos revelan que, desde inicios de la década de 1870, ya circulaban las ideas de cambio y transformación de Darwin, Haeckel y otros autores, con la prensa como principal vehículo de circulación y con las obras de naturalistas e intelectuales (mexicanos y extranjeros) en segunda instancia. Creemos que Molina no fue la excepción y que tuvo acceso a ambos medios, de donde surgieron sus múltiples contactos e intereses con las tesis de evolucionistas, para luego profundizar en las fuentes primarias, como se observa en LGPN.

Del mismo modo, nos parece que existieron redes de comunicación e intercambio de ideas (y publicaciones) evolucionistas entre México, Francia, Inglaterra y EEUU, además de los viajes que la élite intelectual mexicana realizaba por todo el mundo. México no fue un receptor pasivo, ajeno a las discusiones y análisis de diversas nociones evolucionistas; gozó, más bien, de riqueza y pluralidad en las interpretaciones hechas por autores nacionales. En ese sentido, modelos difusionistas que explican la introducción del conocimiento (Basalla, 1967), dejan de lado elementos que interactuaron en la apropiación e interpretación de tesis evolucionistas por parte de autores como Molina Enríquez. Ejemplo de esos elementos son la cultura material (libros, panfletos, instrumentos experimentales y publicaciones que se analizan en LGPN), las redes de colaboración (cargos políticos que desempeñó Molina y sociedades en las que participó) y las geografías locales (discusiones con sus connacionales, como Riva Palacio o Vergara-Lope).

Dichos elementos, ausentes en el difusionismo, permiten entender la circulación del conocimiento como una forma de comunicación (Secord, 2004), donde no hay un proceso lineal, fluido ni uniforme, pero sí interacciones complejas entre diferentes actores, intermediarios y sociedades (Raj, 2021, p.199-204; Silva, Raj, 2016), que nos dan nuevas aproximaciones a la interpretación de Molina sobre las ideas evolucionistas. Esto tiene más sentido cuando aceptamos que el desarrollo de su pensamiento evolucionista ocurrió bajo el contexto histórico y social en el que se encontraba inmerso. Partiendo de la teoría de la recepción de Hans Jauss, sus interpretaciones implicaron una transformación de las ideas, condicionada por diversos contextos socio-históricos, como el de las obras originales, el de las primeras audiencias y el de Molina Enríquez como lector con un papel activo. Los contenidos de estos textos fueron modificados y se imbricaron en un “horizonte de expectativas”, surgido de un proceso que inicia en la recepción simple y pasiva, continúa con la comprensión crítica activa y finaliza con la nueva producción (Jauss, Benzinger, 1970, p.8).

La producción de interpretaciones se vincula con un último hecho que queremos resaltar: así como Molina, hubo miembros de la élite porfirista que esgrimieron apreciaciones y argumentos evolucionistas en torno a los grupos raciales de la población mexicana. Casos interesantes son Francisco Bulnes (1847-1924) y Justo Sierra. Con base en la dieta y en planteamientos evolucionistas, el ingeniero Bulnes (1899, p.5-7) justificó la existencia de tres razas universales: la raza de arroz (asiáticos), la raza de maíz (americanos) y la raza de trigo (europeos). Marichal (2023) y Vargas (2004) sostienen que la herencia biológica y el cambio racial propuestos por Bulnes eran lamarckianos, y por lo tanto las razas podían adquirir atributos a través de la dieta y heredarlos mediante su uso y desuso. En realidad, ese discurso, basado en biología racial y en la inferioridad del maíz frente al trigo, infundió “veneno” al señalar que los enemigos de los pueblos latinoamericanos eran ellos mismos y no Europa o EEUU (Tenorio, 2010). De modo que los inferiores, modificando la dieta, podían convertirse en superiores, pero bajo un contexto evolucionista que permitía alcanzar la fisionomía europea.

Por su parte, Justo Sierra (1902), en *México: su evolución social*, propuso un evolucionismo a partir del progreso en el organismo social. El autor planteó que la sociedad se desarrolla según los principios de variación, lucha por la existencia y selección natural de Darwin, los cuales establecen diferencias biológicas en los humanos que los hacen aptos para la riqueza, el mando o la educación, propiciando su diferenciación en clases sociales (Ruíz, Noguera, Rodríguez, 2015, p.79). Según lo anterior, cada raza, por selección natural, ocuparía un puesto social. Asimismo, Sierra apuntaba a cambios graduales y progresivos para alcanzar la adaptación de la sociedad; proceso que definió como evolución social (Esparza, Ruíz, 2017, p.272). En ese proceso la educación era crucial para la transformación, como lo expresó durante la inauguración de la Universidad Nacional, en 1910: “Cuando el joven sea hombre, es preciso que la Universidad o lo lance a la lucha por la existencia en un campo social superior, o lo levante a las excelsitudes de la investigación científica” (Sierra, 2004, p.23).

Si bien vemos que, para Molina y Sierra, el progreso pasaba por la organización orgánica de la sociedad, con fenómenos similares que explicaban lo natural y lo social, nosotros sostenemos que el pensamiento racial del periodo, a partir de nociones evolucionistas, no fue precisamente el mismo, como hemos analizado previamente. Molina argumentó a favor del mestizaje debido a la biología y resistencia indígena; Bulnes apostó por un discurso anti indígena y por un cambio dietético (menos maíz y más trigo) para europeizar a los mexicanos; y Sierra aseguró que la educación mejoraría a la población, con el trasfondo de selección natural y diversidad racial. Este contraste, entre coetáneos del Porfiriato, ayuda a entender la particularidad de Molina con respecto a su concepción sobre las razas.

## Consideraciones finales

*Los grandes problemas nacionales* es una obra que se encuentra atravesada por diversos argumentos evolutivos y raciales para justificar temas como las fuerzas interiores y exteriores, el desarrollo de las razas, la selección en individuos y en grupos, la superioridad y resistencia indígena, el mestizaje mexicano y la patria ideal.

Las lecturas de distintos naturalistas forjaron en Molina un pensamiento evolucionista y racial. Las ideas evolucionistas le permitieron desarrollar sus tesis sobre el origen y desarrollo de las formas y de las razas, proponer procesos de selección individual y colectiva, y argumentar a favor de la superioridad indígena; mientras las ideas raciales le posibilitaron configurar un sistema mestizante, cuya raza ideal, la mestiza, sería la responsable de sortear los principales obstáculos nacionales.

En este trabajo se muestra que Darwin, Haeckel y, en menor medida, Lamarck influenciaron al positivista. El empleo de citas, el conocimiento de sus libros y teorías, así como el desarrollo de planteamientos evolucionistas en LGPN a partir de estos naturalistas, corroboran dicha influencia. De Haeckel, Molina toma la fuerza formatriz interna para justificar el proceso físico-químico de la vida, así como el equilibrio y la arquitectura de los organismos. Asimismo, le sirven la teoría del carbón y la fuerza vital para respaldar la evolución y la fisiología de los mexicanos, aunque también se apoya sustancialmente en Riva Palacio y Vergara-Lope.

En cuanto a Darwin, su incidencia es amplia y matizada. De alguna manera, el mexicano hace malabares con conceptos procedentes de *El origen* (selección natural, supervivencia del más fuerte, variación o adaptación) y los introduce astutamente en sus largas argumentaciones sobre resistencia, fuerzas externas, efectos ambientales, selecciones individual y colectiva. El ambiente darwinista como agente modificador se aprecia en Molina cuando las condiciones del medio alteran las formas. Su razonamiento es una paráfrasis de lo propuesto en *El origen*: con una previa variación, los organismos más aptos son aquellos que se han adaptado a las condiciones del medio. En la selección individual de las razas, o bien, en los grupos indígenas físicamente superiores, las variaciones ventajosas propiciaron sobrevivir en el ambiente, como sugirió Darwin, y ciertos rasgos se fueron mejorando, poco a poco, según las circunstancias. En este mismo contexto, Molina evoca, entrelineas y a través de la figura preponderante de Haeckel, los planteamientos lamarckianos de uso, desuso y herencia de caracteres, revelando la literatura científica inglesa, alemana y francesa inmersa en LGPN.

La toma de ideas evolutivas de autores naturalistas corresponde a un contexto en el cual disciplinas como la biología estaban en plena conformación. Las premisas de Molina, lejos de ser inoportunas, ayudan a analizar y a entender diversos procesos evolucionistas que se suscitaban en esa época; continuaron, además, con toda una escuela incipiente a finales del siglo decimonónico mexicano, pero vigente en nuestros días: los estudios sobre mestizaje.

Gracias a sus orígenes, a su formación positivista, a la influencia de Riva Palacio acerca de los indígenas, a las lecturas de naturalistas como Haeckel y Darwin, y a su juventud colmada de pasajes donde apreció la adversidad indígena, asoma en Molina un pensamiento evolucionista que no buscaba la erradicación de los pueblos originarios, sino su incorporación a los mestizos mexicanos. Tal pensamiento, huelga decirlo, contrasta con el hispanismo de una época dictatorial y positivista y, más bien, antepone al indígena en el inescapable y necesario mestizaje con los criollos y otros grupos occidentales.

Queda por explorar el papel que tuvieron más naturalistas en el pensamiento de Molina. Casos interesantes serían Wallace o Spencer. Del primero, hay posibilidades de que el abogado lo haya leído en el Instituto Literario del Estado de México; del segundo, se ha comentado la influencia que tuvo en don Andrés para justificar la evolución de las sociedades y alcanzar un perfeccionamiento civilizatorio. Asimismo, el organicismo del filósofo inglés se ajusta a la necesidad de aclarar las partes de todo un cuerpo nacional, para así amalgamar las clases sociales y los grupos raciales del territorio mexicano. No está de más recordar que el darwinismo social se basa en las nociones de Spencer que se trasvasaron del campo biológico al social para justificar las jerarquías de los grupos humanos.

En este escrito hemos analizado y profundizado las ideas evolucionistas de Molina, que a su vez han sido poco examinadas por los estudios históricos de la ciencia. En ese terreno, nuestro trabajo permite vincular posturas historiográficas recientes para realizar futuras investigaciones sobre la circulación del evolucionismo en México. Del mismo modo, aquí se abren nuevas avenidas para explorar la compleja figura de Andrés Molina Enríquez.

## Data Availability

No están en repositorio.

## NOTAS

1 La primera edición fue publicada por la Imprenta A. Carranza e Hijos. Para este estudio, nos valemos de la edición del Instituto Nacional de Estudios Históricos de las Revoluciones de México (2016).

2
Juárez-Barrera y Bueno-Hernández (2017, p.6) apuntan que Molina no adoptó planteamientos de Darwin: “Es claro que estos conceptos novedosos que introduce Molina no provienen de Darwin. No hay ni en *El origen de las especies* ni en *The descent of man* ninguna alusión al respecto”. Los autores se refieren a los conceptos de evolución y selección que aparecen en LGPN para justificar la biología superior del mestizo. Darwin sí que habla de selección y evolución, pero ciertamente no son conceptos idénticos a los manejados por Molina. Nuestra propuesta es que el jurista pudo haber tomado de Darwin estos conceptos y los reinterpretó para sus fines, según los análisis de este trabajo.

3 El empleo del término “raza” en LGPN tiene una connotación amplia: a veces describe cualidades físicas y en ocasiones clases sociales (Bonfil Batalla, 1967, p.225; Magallón, 2003, p.17; Tenorio, 2009, p.43). Es complicado escapar a los significados del término “raza” durante esa época. La creencia en las razas era legítima y peligrosa: facilitó sistemas de dominación basados en color de piel y en características físicas que “implicaban” algún nivel de civilización y de inteligencia. No podemos criticar las etiquetas raciales empleadas por Molina; usaba conceptos de su época y de su contexto. En la actualidad, han cambiado las interpretaciones sobre las razas y se sabe que son inviables biológicamente. Para abundar más, ver Graves y Goodman (2022). Para el caso mexicano, ver García Murcia (2017) y Vera (2019), quienes estudian a las razas desde tres procesos consustanciales: poblamiento de América, antropología criminal y fisionomía indígena.

4 Haeckel readaptó ideas de varios célebres naturalistas. Su libro *The history of creation* (1876) contiene interpretaciones sobre las tesis de Cuvier, Lamarck o Darwin. Los estudios introductorios del darwinismo en México constatan la apropiación de ideas por parte de Haeckel y la influencia del autor en ciertos intelectuales mexicanos**.** Para indagar más, ver Moreno (1984), Ruíz (1987) y Barahona (2009).

5 Sobre el positivismo en México, ver Hale (1991), Priego (2016) y Rodríguez (2024). Para examinar el positivismo en el Instituto Literario del Estado de México, ver Buchanan (1981) y Guevara (2002).

6 Sobre los puestos y nombramientos, ver Basave (2002, p.44-50). Distintos autores coinciden en la falta de una profunda biografía sobre Molina (Kourí, 2009; Krauze, 1998). Sin ser su principal objetivo, Shadle (1994), Kourí (2009), Sánchez (1990) y Basave (2002) recapitulan vida y obra del autor.

7 Indígena o indio, para Molina son sinónimos. Son empleados indistintamente en LGPN. En nuestros análisis sobre Molina prevalecerá la palabra indígena. *Grosso modo*, actualmente los términos tienen connotaciones distintas: mientras uno se refiere a la conquista y a la confusión de América con la India (de allí el gentilicio indio), el otro es político y hace referencia a la cultura de los pueblos originarios. Para más información, ver Bonfil Batalla (1977).

8
Kourí (2009, p.295) explora algunas ideas de Haeckel apropiadas por Molina, como la ley biogenética y el monismo filosófico. Por su parte, Castañeda (2001, p.202) indica que “Molina Enríquez acudió a las ideas de Haeckel para sostener la superioridad de la raza mestiza, pues este admitía que el hibridismo podía representar una fuente de origen de nuevas especies”.

9 En esta y en las demás citas de textos en otros idiomas, la traducción es libre.

10
*El origen de las especies* está mencionado por Molina Enríquez (2016) en las páginas 66 y 449. Los otros libros están mencionados en ediciones francesas: *La descendence de l’homme* (1872) y *De la variation des animaux et des plantes á l’etat domestique* (1868). Estos últimos los comparte como fuentes de otros autores.

11 Se tiene constancia de que, en el Instituto del Estado de México, en 1883, cuando estudiaba allí Molina, existía una biblioteca con 35 libros científicos provenientes de Inglaterra (Guevara, 2006, p.230-231) en los que probablemente estaban los escritos de Darwin. Estos libros y la cátedra de historia natural del Instituto contribuyeron al pensamiento evolucionista de los alumnos. Además, el *Boletín del Instituto* publicó la traducción, hecha por un alumno, de un escrito de Alfred Russell Wallace, con contenido evolucionista. Este dato “confirma el conocimiento vanguardista en ciencias naturales al que tenían acceso los alumnos del Instituto” (Guevara, 2006, p.235). Sería interesante, en otra investigación, averiguar si Molina tuvo acercamientos con este texto de Wallace.

12 Para revisar con escrutinio la teoría del carbón, ver *Morfología general de los organismos* (Haeckel, 1887), donde se examinan fenómenos de locomoción y crecimiento. En el capítulo XIII de *The history of creation* (Haeckel, 1876) también se encuentran elaboraciones sobre la teoría del carbón y la fuerza vital.

13 Al respecto, coincidiríamos con García Murcia (2017, p.155), quien opina que la historia mexicana, para Molina Enríquez, “se explicaba como una interacción de los grupos o razas humanas, en combinación con las características del medio y la alimentación”. Aguilar (2019, p.119-120) menciona que la “vía lamarckiana” fue socorrida para modificar a los mexicanos durante el Porfiriato. El objetivo se lograría mediante “la adopción de una serie de prácticas de alimentación” que propiciaran una herencia genética adecuada. Las élites percibían la comida indígena como inferior y apoyaron los regímenes alimenticios provenientes de Francia. Paralelamente, una eugenesia “neolamarkiana” desarrollaban los médicos mexicanos a finales del siglo XIX, quienes creían que la gonorrea y la sífilis eran hereditarias (Reggiani, 2019, p.138).
